# Integrating Small RNA Sequencing with QTL Mapping for Identification of miRNAs and Their Target Genes Associated with Heat Tolerance at the Flowering Stage in Rice

**DOI:** 10.3389/fpls.2017.00043

**Published:** 2017-01-24

**Authors:** Qing Liu, Tifeng Yang, Ting Yu, Shaohong Zhang, Xingxue Mao, Junliang Zhao, Xiaofei Wang, Jingfang Dong, Bin Liu

**Affiliations:** ^1^Guangdong Key Laboratory of New Technology in Rice BreedingGuangzhou, China; ^2^Rice Research Institute, Guangdong Academy of Agricultural SciencesGuangzhou, China; ^3^Agro-biological Gene Research Center, Guangdong Academy of Agricultural SciencesGuangzhou, China

**Keywords:** miRNA, heat stress, RNA Sequencing, QTL mapping, rice

## Abstract

Although, microRNAs (miRNAs) have been reported to be associated with heat tolerance at the seedling stage in rice, their involvement in heat tolerance at the flowering stage is still unknown. In this study, small RNA profiling was conducted in a heat-tolerant variety Gan-Xiang-Nuo (GXN) and a heat-sensitive variety Hua-Jing-Xian-74 (HJX), respectively. Totally, 102 miRNAs were differentially expressed (DE) under heat stress. Compared to HJX, GXN had more DE miRNAs and its DE miRNAs changed earlier under heat stress. Plant Ontology (PO) analysis of the target genes revealed that many DE miRNAs were involved in flower development. As a parallel experiment, QTL mapping was also conducted and four QTLs for heat tolerance at the flowering stage were identified using chromosome single-segment substitution lines derived from GXN and HJX. Further, through integrating analysis of DE miRNAs with QTLs, we identified 8 target genes corresponding to 26 miRNAs within the four QTL regions. Some meaningful target genes such as *LOC_Os12g42400, SGT1*, and *pectinesterase* were within the QTL regions. The negative correlation between miR169r-5p and its target gene *LOC_Os12g42400* was confirmed under heat stress, and overexpression of miR169r-5p enhanced heat tolerance at flowering stage in rice. Our results demonstrate that the integrated analysis of genome-wide miRNA profiling with QTL mapping can facilitate identification of miRNAs and their target genes associated with the target traits and the limited candidates identified in this study offer an important source for further functional analysis and molecular breeding for heat tolerance in rice.

## Introduction

Rice is the major food for over half of the world population. Therefore, sustainable rice production is very important for the world food security. However, rice farming is always subjected to different abiotic and biotic stresses. Heat stress is one of the major abiotic stresses that significantly affect rice growth and development. The previous study showed that a 7 ~ 8% yield decrease in rice for each 1°C increase in daytime maximum/nighttime minimum temperature from 28/21 to 34/27°C (Baker et al., 1992). Flowering (anthesis and fertilization) is the most sensitive stage to temperature in rice (Satake and Yoshida, 1978; Farrell et al., 2006). Rice plants at anthesis that are exposed to temperatures >35°C for 5 days are sterile and set no seed (Satake and Yoshida, 1978). Exposure to 33.7°C for 1 h at flowering stage could cause IR64 (lowland *indica*) and Azucena (upland *japonica*) significant sterility (Jagadish et al., 2007). It is noteworthy that global warming has exerted a significant effect on rice production. A study conducted by International Rice Research Institute (IRRI) revealed that annual mean maximum and minimum temperatures increased by 0.35 and 1.13°C, respectively, for the period of 1979–2003 at International Rice Research Institute, Manila, Philippines, and grain yields declined by 10% for each 1°C increase in growing-season minimum temperature in the dry season (Peng et al., 2004). Furthermore, a report from the Intergovernmental Panel on Climatic Change (IPCC) predicted that plants would be grown in much warmer environments with the average surface temperature increase of 2.0 ~ 4.5°C by the end of this century (IPCC, 2007). Therefore, it is an urgent task to develop rice variety with heat tolerance to cope with the world climate change. However, heat tolerance in rice is a complex trait controlled by multiple genes (QTLs; Tabata et al., 2007; Ye et al., 2012). Understanding the mechanisms of rice in response to heat stress and identifying the genes associated with heat tolerance are the prerequisite for effective molecular breeding for heat tolerance in rice.

Recently, more and more evidences indicated that microRNAs (miRNAs), a sort of endogenous small non-coding RNAs, play pivotal roles in plant stress responses at the post-transcriptional level (Liu and Chen, 2010). miRNAs exert their functions through negatively modulating the expression of genes by promoting the degradation of target mRNAs or by inhibiting translation. A large number of miRNAs have been demonstrated to function in regulation of plant response to biotic and abiotic stresses, including pathogen invasion (Sunkar et al., 2006; Boccara et al., 2014; Li Y. et al., 2014), nutrient starvation (Fujii et al., 2005; Kawashima et al., 2009, 2011; He et al., 2014), cold (Sunkar and Zhu, 2004; Lv et al., 2010), salt (Song et al., 2013; Zhou et al., 2013), drought (Ni et al., 2013; Song et al., 2013; Fang et al., 2014), and oxidative stresses (Sunkar et al., 2006; Jagadeeswaran et al., 2009). For example, miR319 positively regulates plant response to drought and salinity stress (Zhou et al., 2013). MiR394 negatively regulates salt tolerance but positively regulates drought tolerance in an abscisic acid-dependent manner in *Arabidopsis* (Song et al., 2013). Furthermore, miR826 and miR5090 mediate nitrogen starvation adaptation via regulation of glucosinolate synthesis in *Arabidopsis* (He et al., 2014).

With the rapid development of high throughput sequencing technology in the last few years, a genome-wide search for miRNAs that are responsible for heat stress has been reported in many plants such as wheat (Xin et al., 2010), barley (Kruszka et al., 2014), Chinese cabbage (Yu et al., 2011), Chinese white poplar (Chen et al., 2012), Arabidopsis (Guan et al., 2013; Stief et al., 2014), celery (Li M. Y. et al., 2014), and rice (Jeong et al., 2011). Although, the study of miRNAs in heat tolerance has been made in rice at the seedling stage, a genome-wide identification of miRNAs responsive to heat stress at the flowering stage, the most sensitive stage to temperature and important to grain yield in rice has not been conducted. According to the previous study, many miRNAs express differently in different tissues in rice (Jeong et al., 2011). We reason that there might be different sets of miRNAs in response to heat stress at the seedling stage and the flowering stage in rice, respectively. Thus, to fully understand the mechanism of rice in response to heat stress and identify the potential functional genes useful for molecular breeding, it is worthy to perform a genome-wide profiling of miRNAs at the flowering stage in rice.

Previous genome-wide miRNA profiling experiments have been done using only one genotype. In this study, to better understand the mechanism of miRNA in regulation of heat tolerance in rice, two parallel small RNA profiling experiments were performed at the flowering stage in a heat-tolerant variety Gan-Xiang-Nuo (GXN) and a heat-sensitive variety Hua-Jing-Xian-74 (HJX). We identified the conserved miRNAs in GXN and HJX, respectively. More differentially expressed (DE) miRNAs and earlier expression change of the DE miRNAs were observed in the heat-tolerant variety GXN over the heat-sensitive variety HJX during heat stress and these results may partially explain the difference in heat tolerance between the two genotypes. By integrating genome-wide analysis of DE miRNAs with mapping of QTLs for heat tolerance at the flowering stage in GXN, limited candidate miRNAs and their target genes associated with heat tolerance in GXN were identified. Our data provide new insight into the mechanism of rice in tolerance to heat stress and the limited candidates identified in this study offer an important source for further functional analysis and molecular breeding for heat tolerance in rice.

## Materials and methods

### Plant materials

Two rice varieties that exhibit contrasting sensitivity to heat stress were used for this study: the heat-tolerant variety GXN (*indica*) and heat-sensitive variety HJX (*indica*). Twenty-three chromosome single-segment substitution lines (SSSLs) derived from HJX (recipient) and GXN (donor; Zhang et al., 2004) were used for QTL mapping. Each SSSL contains only one chromosomal segment that is different from HJX. The SSSLs used in the present study are listed in Table S1.

### Estimation of the length of substituted chromosome segment in a SSSL

The lengths of substituted chromosome segments in single segment substitution lines were calculated based on their graphical genotypes (Young and Tanksley, 1989; Hospital, 2002). A chromosome segment flanked by two markers of donor type (DD) is considered as 100% donor type, a chromosome segment flanked by two markers of recipient type (RR) is considered as 0% donor type, and a chromosome segment flanked by one marker of donor type and one marker of recipient type (DR) is considered as 50% donor type. Therefore, the length of DD plus the length of two half DR is considered as the length of a substituted chromosome segment.

### Evaluation of heat tolerance

Thirty plants per line were transplanted in cylindrical plastic pots (11 cm in diameter × 23 cm in height). Single plant was planted in one pot. Each cylindrical plastic pot was filled with 1.00 kg of dry fine soil and 0.64 g of compound fertilizer (N-P_2_O_5_-K_2_O, N:P:K = 12:18:15). After the transplanted seedlings established and grew up to the tillering stage, each pot was applied with 0.33 g of compound fertilizer. Extra tillers were removed, leaving two tillers per plant to avoid overcrowding and to ensure better growth. At the heading stage, when the panicle exserted about 2 cm from the auricle, the plant was used for heat stress treatment. Twenty uniform plants per line were selected and divided into two groups with 10 plants each. The two groups of plants were transferred into two Conviron PGV36 growth chambers (Controlled Environments Ltd., Winnipeg, Canada), respectively. The photon flux density and relative humidity in the growth chambers were 200 μmol m^−2^ s^−1^ (6:00–18:00) and 75 ± 5%, respectively. The temperature of the growth chamber for control was 28.0/22.0°C (day/night), while the average temperature of the growth chamber for heat stress was 31.0°C. The concrete day and night temperature parameters for heat treatment were shown in Figure S1. After treated for 7 days in the growth chambers, the plants were taken back to the screen house for normal growth until maturity. At maturity, the following traits of tested plants were measured: total number of spikelets per plant (TSP) and the number of filled spikelets per plant (FSP). Spikelet fertility was expressed as SFP = FSP/TSP × 100%. The heat tolerance index (HI) was used to evaluate heat tolerance, HI = heat treated SFP/control SFP. Three independent experiments were conducted.

### QTL analysis

The SSSLs derived from the heat-tolerant variety GXN (donor parent) and the heat-sensitive variety HJX (recurrent parent) were used to detect QTLs for heat tolerance. Theoretically, there is only a single segment difference between each SSSL and HJX. The detection of QTL for heat tolerance and its effect were based on the difference in heat tolerance between each SSSL and HJX. *T*-test was used to analyze the significance of difference using the program *SPSS* 12.0. A QTL was declared when the significance level was <0.05. According to Eshed and Zamir (1995), QTL additive effect = (phenotype of SSSL − phenotype of HJX)/2; QTL additive effect percentage = additive effect/phenotype of HJX × 100%.

### Heat stress treatment and sampling

Mature seeds of GXN and HJX were incubated at 49°C for 4 days prior to germination, and then sown in pots with fine soil. The growth condition of the rice plants were the same as described in the evaluation of heat tolerance with some modifications. The control growth chamber was maintained at 27°C with a 12 h light/12 h dark photoperiod, and the growth chamber for heat treatment was maintained at 38°Cwith a 12 h light/12 h dark photoperiod. Panicle samples from both heat-treated and control plants were collected at 1, 6, and 24 h after heat treatment. Three biological replicates were conducted in the present study. All collected samples were immediately frozen in liquid nitrogen and stored at −70°C.

### Total RNA isolation and small RNA deep sequencing

Samples of three biological replications were pooled together for small RNA sequencing. Total RNA from heat treated and control samples of GXN and HJX was isolated with Trizol (Invitrogen, Carlsbad, CA) according to the manufacturer's instruction. Small RNA library preparation and sequencing were performed with Solexa Sequencing Technology (ANNOROAD, Beijing, China). For construction of small RNA libraries, total RNA was fractionated by 15% polyacrylamide gel electrophoresis, and small RNAs in the range of 18–30 nt were purified. After dephosphorylation and ligation of a pair of Solexa adaptors to the 5′ and 3′ ends, sRNAs were then reverse-transcribed and amplified by PCR. The raw sequencing data has been submitted to NCBI (http://www.ncbi.nlm.nih.gov/) with accession number SRX834694.

### Characterization of known miRNAs from the sequencing data

All sequenced reads were mapped to the *Oryza sativa* genome (MSU7), using the Bowtie program (Li and Durbin, 2009) with version 1.0.0 (-p 16, -f, -n 1, -e 80, -l 18, -a, -m 5, –best, –strata). Only reads less than five perfect matches to the genomic positions were reported. Reads were then searched against miRNA database (miRbase v20) for perfect matches using the miRDeep2 program (Friedländer et al., 2012) with version 2_0_0_5 (-t Rice) and defined miRNAs as “confirmed” when the mature sequence was observed 10 times or more in either library.

### Identification of differentially expressed miRNAs and functional analysis of their targets

All reads were normalized as transcripts per million (TPM) according to the total sequenced reads in each sample. miRNAs with log_2_ Fold change ≥ 1.0 or log_2_ Fold change ≤ −1.0, *P* ≤ 0.05, and FDR ≤ 0.05 were considered to be significantly differentially expressed. Fold change = |TPM ratio| (treatment/control). Fisher's exact test was used to calculate the *P*-value and false discovery rate (FDR) was estimated using Benjamini and Hochberg method in R package. The psRobot_tar with parameters (-ts 2, -gl 10) in program psRobot (version 1.2; Wu et al., 2012) version 1.2 was used for target gene prediction and an online server of psRNATarget (Dai and Zhao, 2011) (http://plantgrn.noble.org/psRNATarget/) was also used for target gene prediction with expectation range 2.0–3.0 and other default parameters when no results were predicted by using psRobot. PlantGSEA (Yi et al., 2013) was used for enrichment analysis of biological processes in Gene Ontology and Plant Ontology. KEGG pathway analysis was carried out on KEGG website (http://www.genome.jp/kegg/).

### Quantitative real-time polymerase chain reaction

Real-time quantification of microRNAs by stem-loop RT-PCR was performed as described by Chen et al. (2005) and Varkonyi-Gasic et al. (2007). RNA RT reactions were performed using the primescript™ RT reagent kit (Takara, Japan). Real-time PCR was carried out using the SYBR Premix Ex Taq™ kit (Takara, Japan), following the manufacturer's instructions, on a Biorad CFX Connect Real-Time System. The 5srRNA and *EF1*α were used as endogenous normalized genes for miRNA and mRNA, respectively. All reactions were run in triplicate. Primers used to amplify the selected genes are listed in Table S2.

### 5′ RLM-RACE

Total RNA was extracted from rice panicles. Poly(A)^+^ mRNA was subsequently enriched using the Oligotex mRNA mini Kit (Qiagen, Germany). 5′ rapid amplification of cDNA ends (5′ RACE) was carried out with GeneRacer Kit (Invitrogen, America) as described by Sunkar et al. (2005). The first round PCR was performed with GeneRacer 5′ primer and gene-specific primer, followed by second round PCR with Gene-Race 5′ nested primer and gene-specific nested primer. The PCR products were gel-purified, cloned, and sequenced. The primers used in this study were shown in Table S2.

### Development of miR169r-5p transgenic plants and evaluation of heat tolerance of transgenic plants

For the *Ubi:miR169r-5p* construct, the precursor sequence of miR169r-5p was amplified from heat-tolerant rice variety GXN by PCR using the following primers: forward primer, 5′- GGTCAACCCAAATAAGCAAG -3′; reverse primer, 5′- GAATAATACAGTGTAGCCATAGAGG -3′. The DNA product was inserted into pOX which harbors an ubiquitin promoter, and the resultant vector was electroporated into *Agrobacterium tumefaciens EHA105*. Rice transformation was performed as described by Toki et al. (2006). Evaluation of heat tolerance of the transgenic plants was the same as indicated above, the transgenic plants were treated by heat stress for 4 days and then taken back to the screen house for normal growth.

## Results

### Performance of the two selected rice genotypes under heat stress

To screen for rice varieties with contrasting heat tolerance at the flowering stage for this study, we have evaluated the heat tolerance of rice germplasm from 11 countries at the flowering stage. Our results showed that GXN, an *indica* variety from China exhibited strong heat tolerance, whereas HJX, an *indica* variety from China was sensitive to heat stress (Figure S2). After heat treatment at the flowering stage, the spikelet fertility of GXN and HJX was 55.8 and 15.3%, respectively (Table 1). The heat tolerance index (HI) of GXN is 0.84, much higher than that of HJX (0.20) and similar to N22, a well-known heat-tolerant *indica* landrace variety (Jagadish et al., 2010), with a HI of 0.85 in the present study (Table 1). Thus, GXN is a strong heat-tolerant variety while HJX is a heat-sensitive variety and their contrasting phenotypes in heat tolerance make them to be ideal materials for exploring the functional genes and molecular mechanisms of heat tolerance in rice.

**Table 1:** **The heat tolerance indexes of GXN, HJX, and N22**.

**Variety**	**Spikelet fertility (%)^a^ control**	**Spikelet fertility(%)^a^ heat stress**	**Heat tolerance index**
HJX	76.6 ± 3.84	15.3 ± 0.91	0.2 ± 0.01
GXN	66.4 ± 4.97	55.8 ± 6.11	0.84 ± 0.09
N22	68.7 ± 6.37	58.67 ± 3.86	0.85 ± 0.06

a *The value is the mean of 10 plants*.

### Genome-wide analysis of small RNAs in heat treated and untreated GXN and HJX at the flowering stage

To identify the miRNAs at flowering stage in heat treated and untreated GXN and HJX, rice plants of the two genotypes at the flowering stage were subjected to heat stress treatment for 1, 6, and 24 h, respectively. Small RNA libraries were constructed from panicle tissues in the heat-treated and untreated rice plants at the given time points. In total, 12 libraries were constructed and subjected to high-throughput sequencing analysis. After trimming the adaptor sequences, total sequencing reads of the 12 libraries ranged from 22.6 to 29.6 million, with the distinct (unique) reads that perfectly matched the rice genome ranged from 4.6 to 8.1 million (Table S3). Reads ranged in size from 18 to 30 nt, with peaks at 21 and 24 nt (Figure S3), consisting with the previous analysis of small RNAs at the seedling stage in rice (Jeong et al., 2011).

Overall, we detected the expression of 446 known miRNAs representing 182 families (Table S4). Among them, 412 miRNAs belonging to 152 families (Table S4) were expressed both in GXN and HJX. Nineteen miRNAs belonging to 17 miRNA families were expressed specifically in GXN, whereas 15 miRNAs belonging to 13 miRNA families were expressed specifically in HJX (Table S5). Both in GXN and HJX, the most abundant expressed miRNA was miR396f-5p, with the reads reached to 958,958 in the library of GXN-1hT (Table S4). Interestingly, 42 miRNAs showed significantly higher basal level (*P* ≤ 0.05 and log_2_ fold change ≥ 1.0) in GXN than that in HJX in at least one of the three comparisons before heat stress (Table S6). The 42 miRNAs include those miRNAs belonging to the highly conserved miRNA families such as miR160, miR166, miR167, and miR168.

### The expression patterns of the heat responsive miRNAs in GXN and HJX

**Figure 1:**
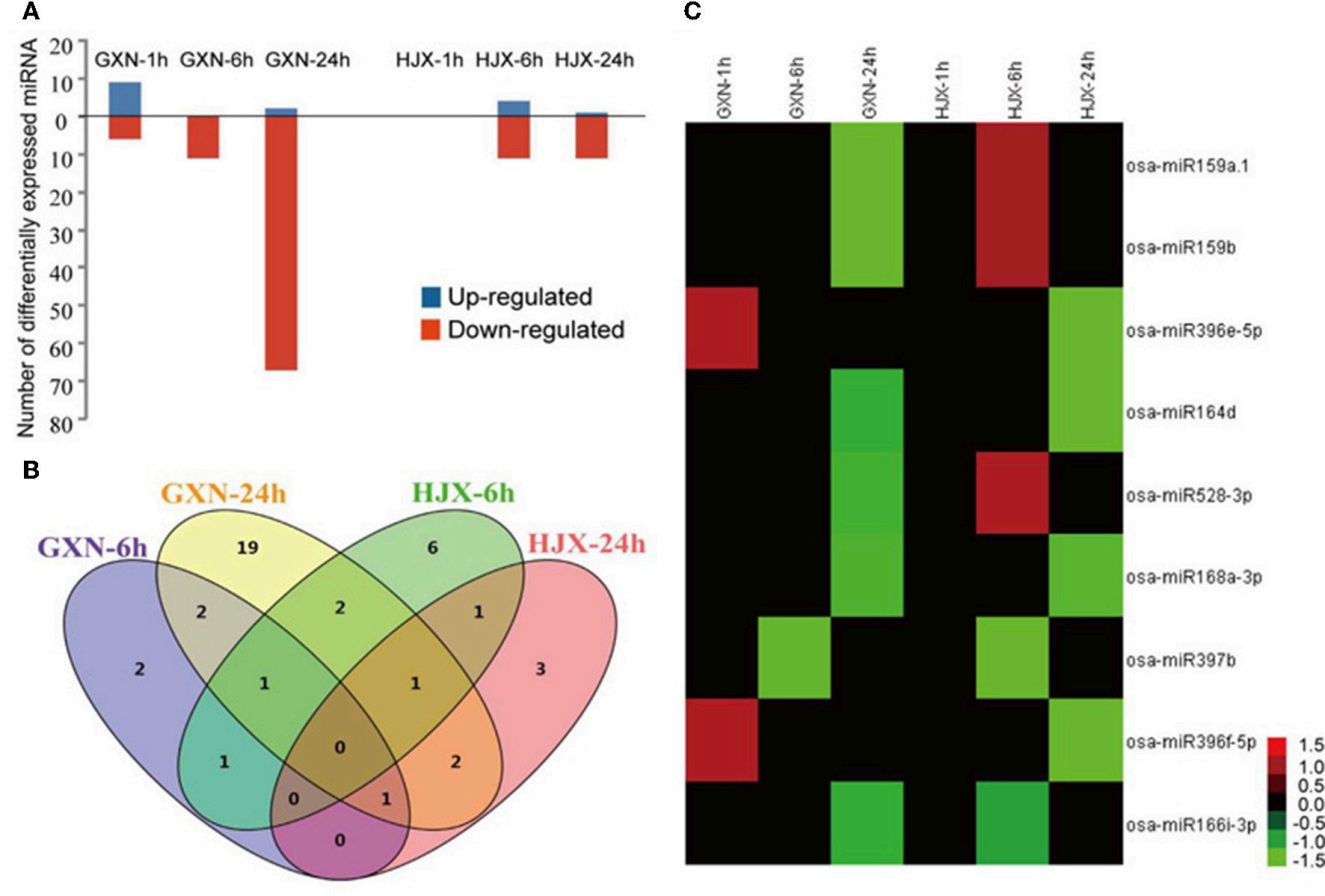
**The expression patterns of 102 differentially expressed (DE) miRNAs (log_**2**_ fold change ≥ 1.0 or log_**2**_ fold change ≤−1.0; ***P*** ≤ 0.05 and FDR ≤ 0.05) under heat stress. (A)** The number of miRNAs that were up-regulated or down-regulated in GXN and HJX under heat stress. **(B)** Number of DE miRNA families in four sequencing libraries. Venn diagram was created using VENNY. **(C)** Expression patterns of 9 DE miRNAs under heat stress both in GXN and HJX.

Overall, 102 miRNAs were differentially expressed (DE) (log_2_ fold change ≥ 1.0 or log_2_ fold change ≤ −1.0, *P* ≤ 0.05, and FDR ≤ 0.05) afterheat stress treatment in GXN and HJX (Figure 1A, Table S7). They could be classified into four categories: (1) co-down-regulated miRNAs in both GXN and HJX; (2) co-regulated miRNAs but showed opposite expression patterns between GXN and HJX; (3) DE miRNAs specifically identified in GXN; (4) DE miRNAs specifically identified in HJX (Table S7). A total of 85 miRNAs were differentially expressed in GXN in at least one comparison, and 26 DE miRNAs were identified in HJX (Table S7). Fifteen miRNAs were differentially expressed at 1 h after heat stress in GXN, whereas no miRNA was differentially expressed at 1 h after heat stress in HJX (Figure 1A). These observations indicated that there were more DE miRNAs and the response of miRNAs to heat stress was earlier in the heat-tolerant variety GXN compared to the heat-sensitive variety HJX.

Among the 102 DE miRNAs, 9 miRNAs were observed both in GXN and HJX (Figure 1B, Table S7). Four of these miRNAs (miR164d, miR166i-3p, miR168a-3p, and miR397b) exhibited the same suppressed expression pattern after heat stress in both of the two rice varieties (Figure 1C), suggesting a persistent regulatory role of these miRNAs in heat tolerance. Furthermore, it is noteworthy that among the 102 DE miRNAs, only 14 of them were up-regulated after heat stress, including the miRNAs which were firstly up-regulated but then down-regulated during heat stress (Table S7). The other DE miRNAs were uniformly down-regulated after subjected to heat stress, indicating that most DE miRNAs were under-presented under heat stress at the flowering stage in rice.

### Target prediction and functional classification of heat responsive miRNAs

**Figure 2:**
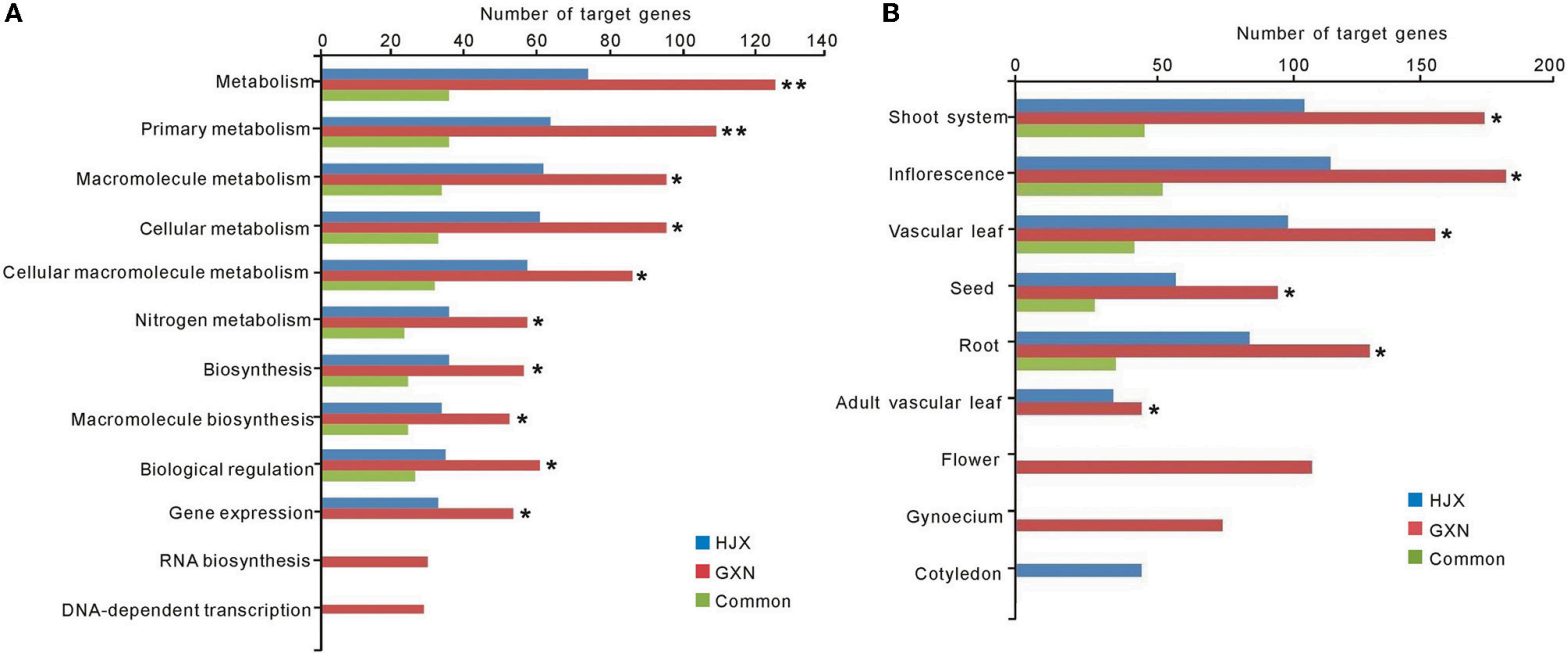
**GO and PO slims of functional categorization of the predicted targets of 102 DE miRNAs**. ^**^ and ^*^ indicate significant differences in the number of target genes between GXN and HJX at *P* ≤ 0.01 and *P* ≤ 0.05, respectively. **(A)** GO enrichment analysis of the predicted targets of 102 DE miRNAs. **(B)** PO enrichment analysis of the predicted targets of 102 DE miRNAs.

DE miRNAs have the potential to regulate target transcripts to exert their functions in rice. To understand the functions and their potential regulatory roles of DE miRNAs in heat tolerance, we searched for the putative targets of the 102 DE miRNAs and performed Gene Ontology (GO) enrichment analysis of these predicted targets. Totally, 446 target genes were identified for the 102 DE miRNAs (Table S8). The GO enrichment analysis suggested that metabolism, biosynthesis, and biological regulation were the most significant biological processes (Figure 2A, Table S9). Moreover, these significantly related biological processes are common between the two rice varieties, further suggesting the important roles of these miRNAs in heat tolerance. However, as shown in Figure 2A, the number of the target genes involved in these biological processes in GXN is much more than that in HJX. On the other hand, some biological processes such as RNA biosynthesis and DNA-dependent transcription are GXN-specific (Figure 2A).

**Figure 3:**
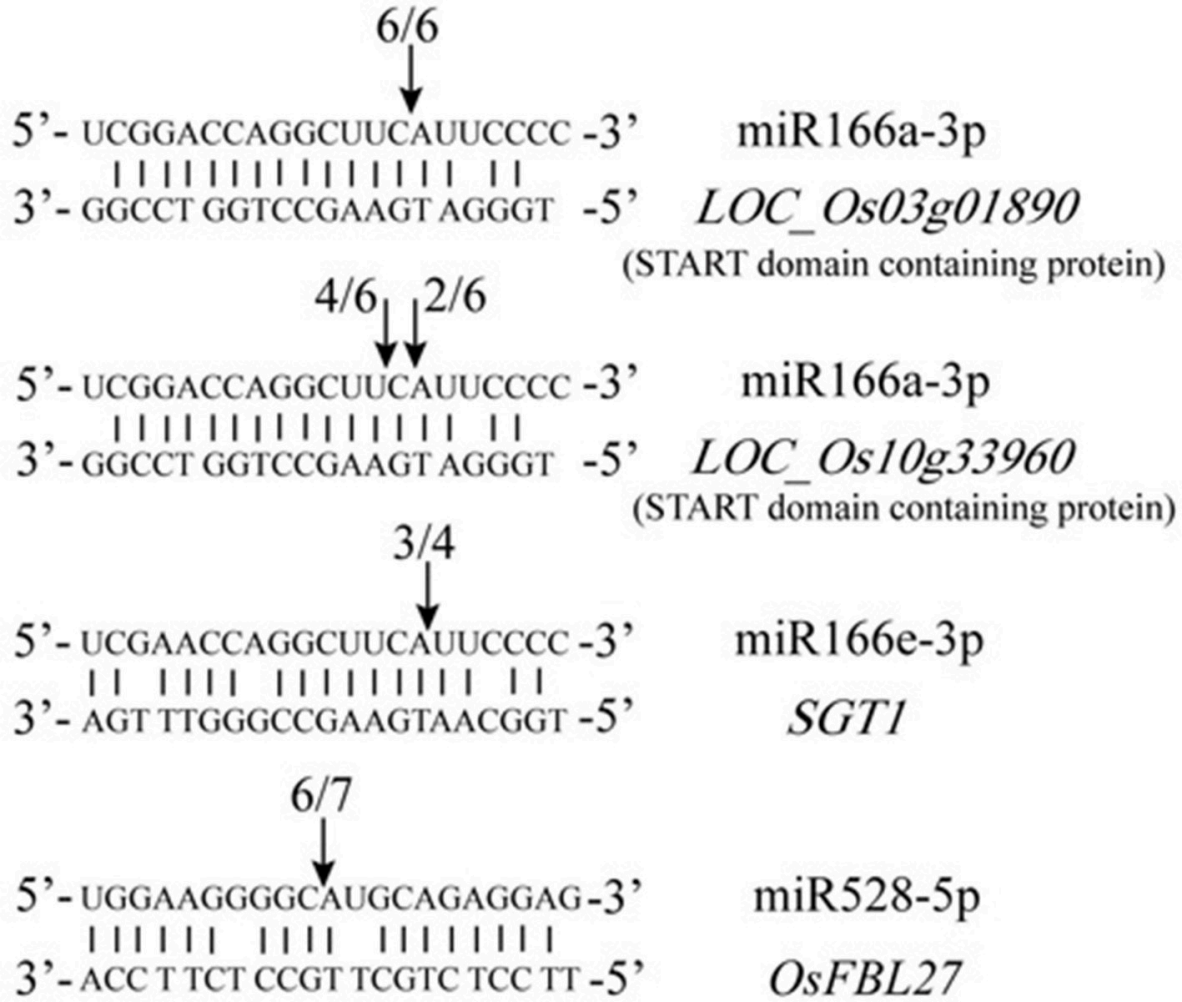
**Validation of predicted target genes by modified RLM 5′-RACE**. The arrows indicate the cleavage sites. The number of cloned RACE products that were sequenced is shown above each sequence.

To further understand their functions of the DE miRNAs identified in this study, PO (Plant Ontology) enrichment analysis of the putative targets of the 102 DE miRNAs was performed. The results showed that among the nine tissues tested, the number of target genes in six tissues in GXN was significantly more than that in HJX (Figure 2B, Table S10). Particularly, “inflorescence”and “shoot system” had the largest number of target genes (Figure 2B). In addition, the target genes in “flower” and “gynoecium” were GXN specific (Figure 2B). Among the miRNAs involved, the previously identified heat responsive miRNA miR166 (Xin et al., 2010) was included. In addition to *HD-ZIP* transcription factors, we identified that miR166a-3p is also predicted to target other two START domain containing proteins which are speculated to be involved in gynoecium development (Figure 3). RLM-RACE validated that these genes could be successfully cleaved by miR166a-3p. These results imply that miR166-mediated cleavage of the two target mRNAs might be involved in gynoecium development and heat tolerance at the flowering stage in rice.

### Validation of RNA sequencing data in this study using quantitative RT-PCR

**Figure 4:**
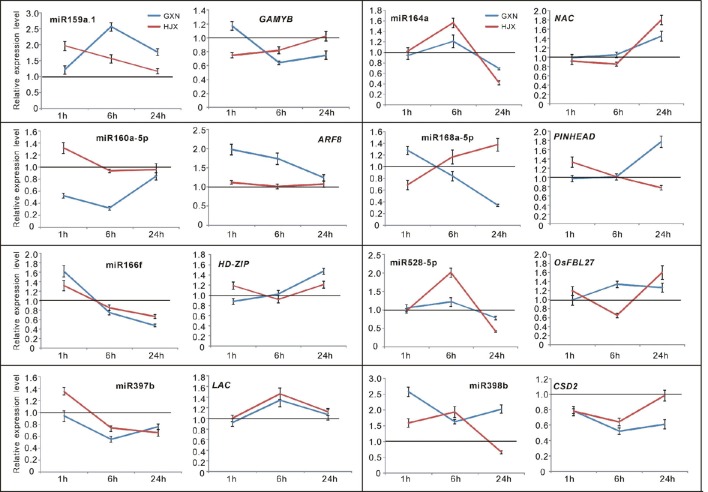
**Time-course expression profiling of the selected miRNAs and their target genes at 1, 6, and 24 h after heat stress treatment at the flowering stage in both heat-tolerant variety GXN and heat-sensitive variety HJX using RT-PCR**. The expression of control plants without heat stress was set to “1.0” at different time points, and was marked by the black line. Bars represent means (three replicates) ±standard derivation (SD).

**Figure 5:**
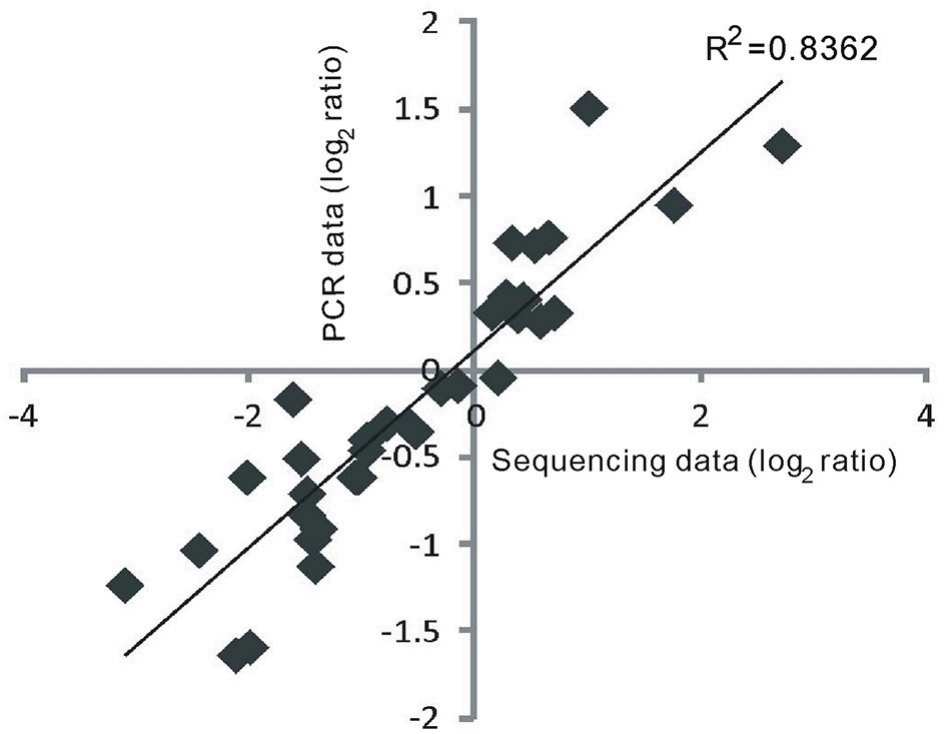
**The results of quantitative RT-PCR showed an excellent concordance with the sequencing data**. The correlation coefficient was calculated by the linear regression.

To confirm the authenticity of the sequencing results, eight DE miRNAs were selected for expression analysis using quantitative RT-PCR (qRT-PCR). The eight miRNAs (miR159a.1, miR164a, miR160a-5p, miR166f, miR168a-5p, miR397b, miR398b, and miR528-5p) all have been reported to be involved in responses to heat stress or other abiotic stresses in plants (Xin et al., 2010; Zhou et al., 2010; Jeong et al., 2011; Chen et al., 2012; Guan et al., 2013; Fang et al., 2014; Stief et al., 2014). The quantitative RT-PCR assays revealed that the selected DE miRNAs exhibited different or the same expression patterns in GXN and HJX (Figure 4). The results showed an excellent concordance with the sequencing data (Figure 5; *R*^2^ = 0.8362). As expected, the transcription of the target genes was negatively correlated with the abundance of their corresponding miRNAs (Figure 4). Thus, these results together indicate the reliability of the RNA sequencing data in the present study.

### Integrating differential expression analysis of miRNAs with QTL mapping to identify miRNAs and their target genes associated with heat tolerance in rice

To cut down the number of candidate miRNAs and their target genes associated with heat tolerance, we performed QTL mapping and integrating QTL mapping with analysis of differential expression of miRNAs. Here, we used chromosome single-segment substitution lines (SSSLs) for identification of QTLs associated with heat tolerance. The SSSLs were developed from GXN (donor) and HJX (recurrent parent; Zhang et al., 2004). Through evaluation of the heat tolerance of the SSSLs and their recurrent parent at the flowering stage, we identified four QTLs for heat tolerance on chromosomes 3, 6, 8, and 12 across three independent experiments, designated as *qHT-3, qHT-6, qHT-8, qHT-12*, and they accounted for 19.32, 30.71, 51.67, and 14.47% of phenotypic variation, respectively (Table 2). These effective alleles are all from the heat-tolerant variety GXN. We looked for those DE miRNAs whose target genes are located in the QTL regions. We found that 8 target genes of the 26 DE miRNAs belonging to 6 miRNA families were identified in the four QTL regions (Table 3).

**Table 2:** **Heat-tolerant QTLs identified at the flowering stage**.

**QTL**	**Chr**	**Marker intervals**	**Length (Mb)**	Additive effect^*^	Additive effect percentage^*^(%)
*qHT-3*	3	RM227-RM565-RM148-End	1.03	3.59	19.32
*qHT-6*	6	RM190-RM587-RM510-RM225-RM217-RM50	1.80	5.60	30.71
*qHT-8*	8	RM404-RM339-RM515-RM223-RM531-RM210-RM149	6.86	9.24	51.67
*qHT-12*	12	RM235-RM17-PSM193-End	0.96	2.72	14.47

* *The value is the mean of three independent experiments*.

**Table 3:** **Differentially expressed miRNAs with target genes located in the QTL regions**.

**QTL**	**miRNA**	**ID of target genes**	**Target gene description**
*qHT-6*	osa-miR528-3p	*LOC_Os06g07530*	Retrotransposon protein, putative
*qHT-8*	osa-miR5794	*LOC_Os08g31390*	Retrotransposon protein
	osa-miR166a/c/e-5p	*LOC_Os08g33630*	UPF0016 domain containing protein
	osa-miR166e-3p	*LOC_Os08g34740*	SGT1 protein
	osa-miR3980a/b-3p	*LOC_Os08g34900*	Pectinesterase
*qHT-12*	osa-miR319a-3p.2-3p	*LOC_Os12g42190*	Transposon protein
	osa-miR319b	*LOC_Os12g42190*	Transposon protein
	osa-miR169a/h/j/k/l/m	*LOC_Os12g42400*	Nuclear transcription factor Y subunit
	osa-miR169i-5p.1	*LOC_Os12g42400*	Nuclear transcription factor Y subunit
	osa-miR169r-5p	*LOC_Os12g42400*	Nuclear transcription factor Y subunit
	osa-miR166a/b/c/d/e/i/j-3p	*LOC_Os12g43900*	Retrotransposon protein, putative
	osa-miR166f	*LOC_Os12g43900*	Retrotransposon protein, putative

**Figure 6:**
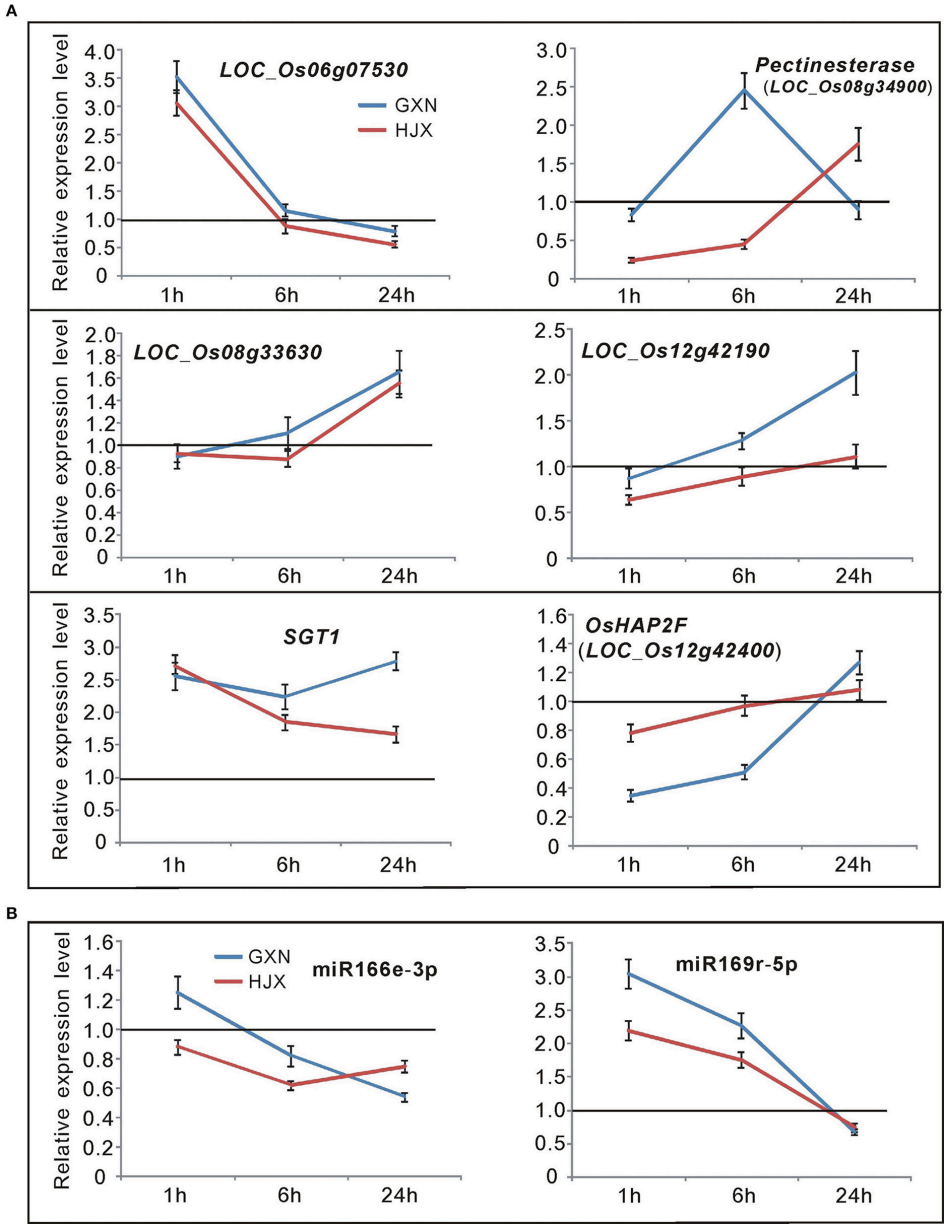
**Time-course expression profiling of the candidate miRNAs and their target genes (in the QTL regions) at 1, 6, and 24 h after heat stress treatment at the flowering stage in GXN and HJX**. The expression of control plants without heat stress was set to “1.0” at different time points, and was marked by the black line. Bars represent means (three replicates) ±SD. **(A)** Time-course expression profiling of 6 target genes in the QTL regions. **(B)** Time-course expression profiling of miR166e-3p and miR169r-5p.

To find the most possible target genes involved in heat tolerance in GXN among these 8 genes within the QTL regions, the transcription levels of the 8 genes in GXN and HJX both before and after heat stress were analyzed using qRT-PCR. The results (Figure 6A) revealed that *LOC_Os08g31390* and *LOC_Os12g43900* were not expressed. *LOC_Os06g07530* and *LOC_Os08g33630* exhibited similar expression patterns both in GXN and HJX. However, *LOC_Os08g34740* (*SGT1*) and *LOC_Os08g34900*, (*pectinesterase*) within *qHT-8, LOC_Os12g42190* and *LOC_Os12g42400* within *qHT-12* all showed differential expression under heat stress between GXN and HJX, suggesting their probable roles in heat tolerance. MiR169 and its target gene *LOC_Os12g42400* (*Os12g0618600* in RAP locus) have been reported to be ABA-responsive (Tian et al., 2015) and it has been well-known that ABA plays a pivotal role in adaptive responses to various abiotic and biotic stresses (Lee and Luan, 2012). *SGT1* (the target of miR166e-3p; Figure 4) can bind to heat shock proteins (HSPs) such as HSP90 and HSP70 and has been demonstrated as a positive regulator in plant and mammalian cells for the response to thermal stress (Arya et al., 2007; Mayor et al., 2007; Zabka et al., 2008; Shirasu, 2009; Prus and Filipek, 2011). Transcription analysis revealed that miR169r-5p and miR166e-3p exhibited negative correlation expression patterns with their target genes (*SGT1, LOC_Os12g42400*) under heat stress (Figure 6B). Furthermore, the signaling pathway analysis of the target genes of the DE miRNAs through KEGG (http://www.genome.ad.jp/kegg/) indicated that the gene *pectinesterase* (target of miR3980a/b-3p) within *qHT-8* was involved in starch and sucrose metabolism which is related to pollen fertility and grain filling under heat stress (Wang et al., 2014).

### miR169r-5p overexpressing plants showed enhanced heat tolerance at the flowering stage in rice

**Figure 7:**
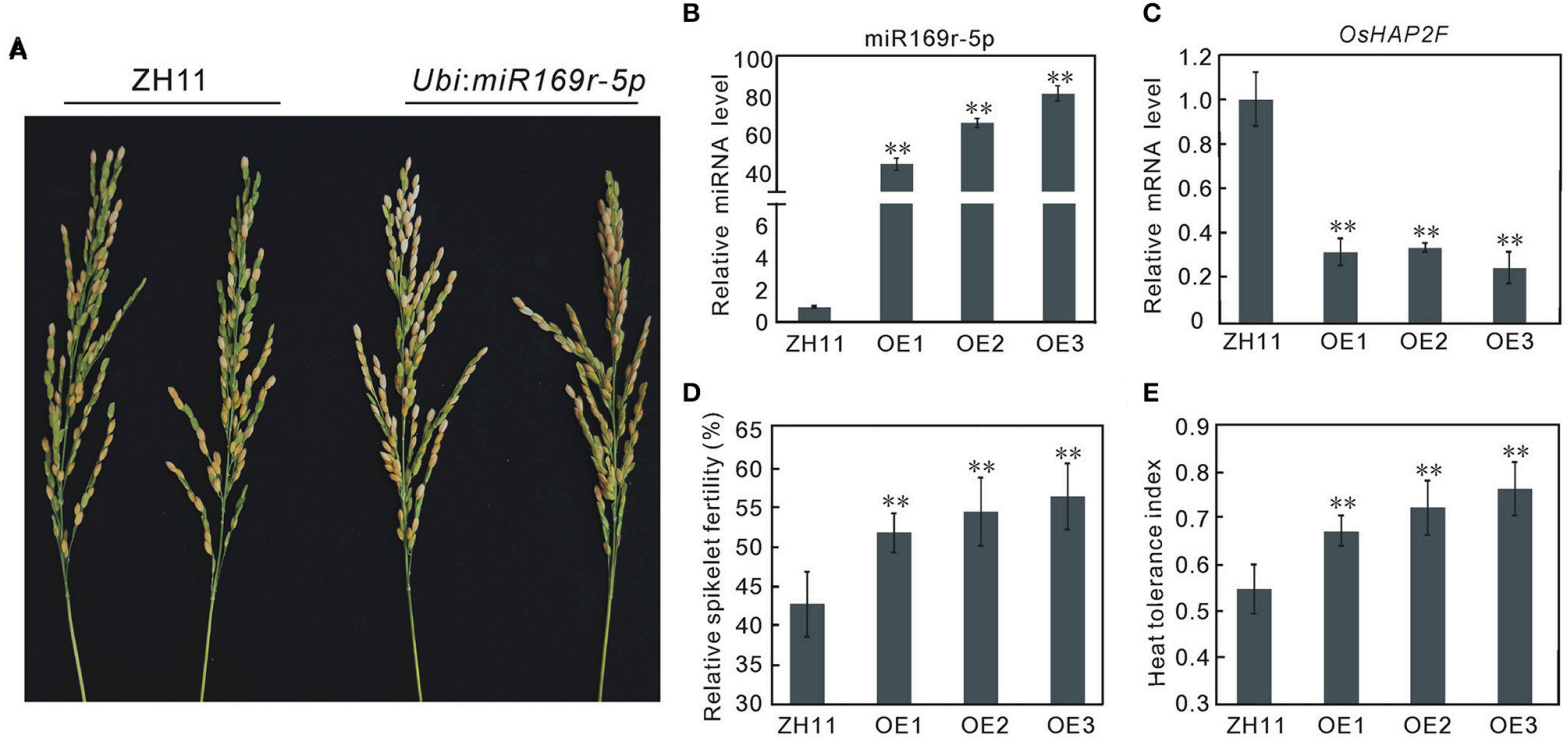
**Overexpression of miR169r-5p enhances heat tolerance at the flowering stage in rice. (A)**, Spikelet fertility of ZH11, the wild type plants and miR169r-5p overexpressing plants (*Ubi: miR169r-5p*) after heat stress at the flowering stage. **(B)** Transcription analysis of miR169r-5p in the wild-type ZH11 and miR169r-5p overexpressing (OE) plants by quantitative RT-PCR. Error bars indicate the SD from three biological replicates. **(C)** Transcription analysis of the target gene *OsHAP2F* (*LOC_Os12g42400*) in ZH11 and transgenic plants by quantitative RT-PCR. Error bars indicate the SD from three biological replicates. **(D)** Relative spikelet fertility of ZH11 and transgenic plants under heat stress. Bars represent means (two replicates) ±SD (*n* ≥ 10). **(E)** Relative heat tolerance indexes of ZH11 and transgenic plants under heat stress. Bars represent means (two replicates) ±SD (*n* ≥ 10). Asterisks indicate statistically significant differences compared with ZH11 (*t*-test, ^**^P < 0.01).

Based on its possible role in heat tolerance and novelty, we selected miR169r-5p for further functional confirmation. The transgenic rice plants constitutively overexpressing miR169r-5p were produced in *Zhonghua11* (ZH11), which is sensitive to heat stress at the flowering stage. The miR169r-5p overexpressing (OE) plants showed no morphological changes and were fertile. Three independent transgenic lines (OE1, OE2, and OE3) with higher miR169r-5p expression were identified by miRNA qRT-PCR compared to the wild-type ZH11 plants (Figure 7B). Consistently, the expression of its target gene *LOC_Os12g42400* was reduced to <35% of that in the control plants (Figure 7C). The spikelet fertility of the transgenic plants ranged from 53.23 to 58.06% (average of 55.79%), significantly higher than that of ZH11 plants (42.50%) (Figures 7A,D). Similarly, the heat tolerance indexes of transgenic plants were also significantly higher than that of ZH11 plants (Figure 7E).

To gain further insight into the mechanisms of the enhanced heat-tolerance of miR169r-5p overexpressing plants, the transcripts of four HSPs that have been reported to be involved in response to heat stress (Qin et al., 2015) were analyzed in the transgenic lines and control plants both before and after heat stress. The results showed that heat stress treatment significantly induced the transcription of these four HSPs both in the control and transgenic plants. However, the transcription levels of the four HSPs were all higher in miR169r-5p overexpressing plants than in control plants (Figure S4), further supporting the positive role of miR169r-5p in heat tolerance.

## Discussion

### miRNAs are involved in heat tolerance at the flowering stage in rice

**Figure 8:**
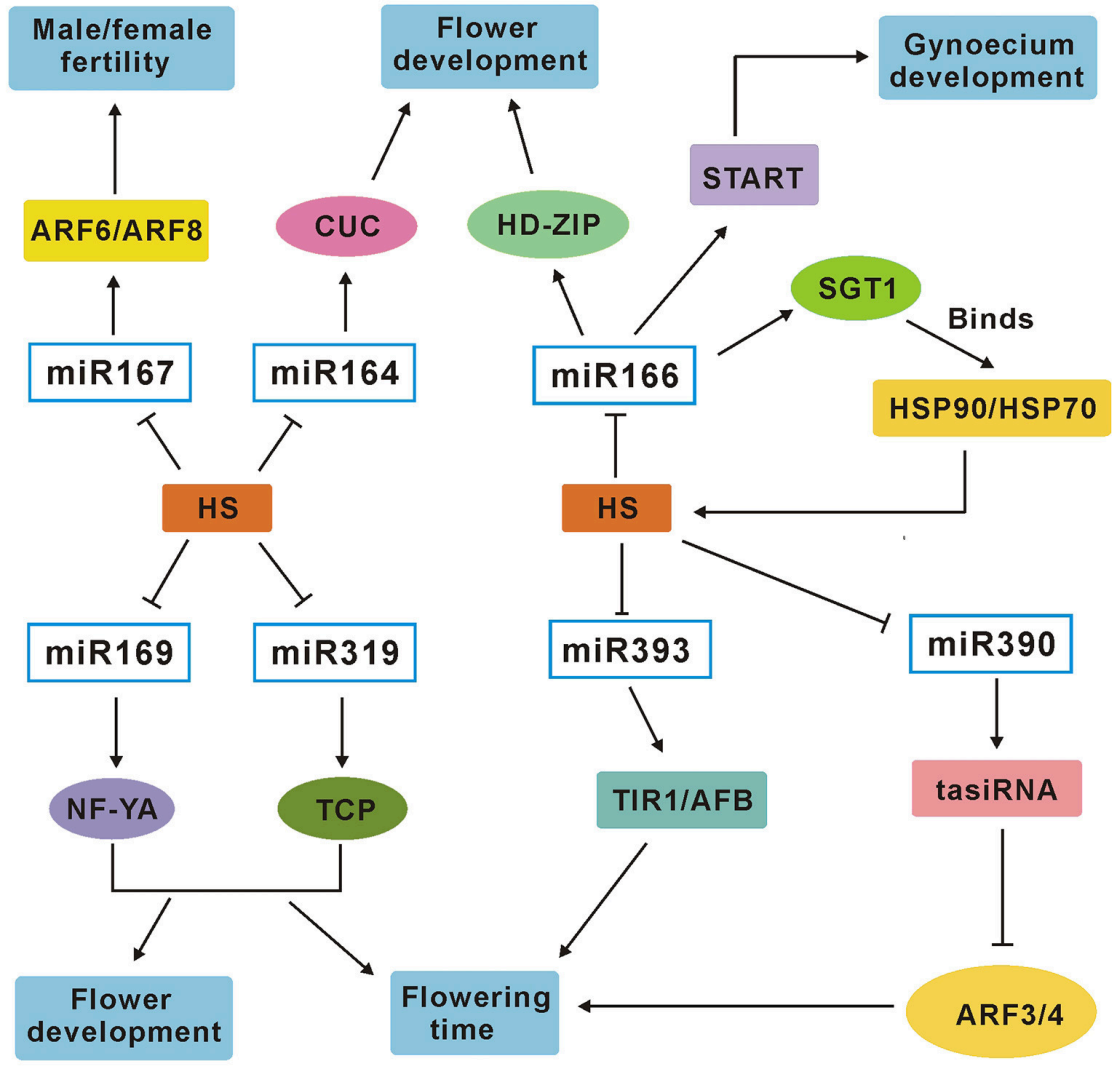
**A miRNA–target genes network module involved in heat tolerance and flower development or fertility in rice plants**. Slim arrows show stimulative effects in the pathway; Nail shape represents repression. HS, heat stress; START, START domain containing protein.

In the past few years, miRNAs have been validated to play crucial roles in biotic and abiotic stresses at the post-transcriptional level by targeting mRNAs for cleavage or repressing translation in many plants (Sunkar and Zhu, 2004; Sunkar et al., 2006; Song et al., 2013; Zhou et al., 2013). Recent studies have also uncovered the complexity of miRNAs in regulation of plants in responses to heat stress using high-throughput sequencing technology and demonstrated that miRNAs may function as important modulators in heat acclimation (Xin et al., 2010; Jeong et al., 2011; Yu et al., 2011; Chen et al., 2012). As one of the most important crops, rice plant is significantly affected by high temperature stress. The previous study has demonstrated that miRNAs play important role in heat tolerance at the seedling stage in rice (Jeong et al., 2011). However, genome-wide analysis of rice heat stress–regulated miRNAs has not been conducted at the flowering stage, which is very important for rice yield and is the most sensitive stage to heat stress (Matsui and Omasa, 2002; Prasad et al., 2006). In the present study, two parallel small RNA profiling experiments were performed at the flowering stage using Illumina sequencing technology in both heat-tolerant variety GXN and heat-sensitive variety HJX. The small RNA expression was monitored at 1, 6, and 24 h. Overall, 102 miRNAs were identified with at least two-fold change during heat stress in the present study. Compared with the previous studies, 63 of the 102 DE miRNAs except for miR3980 have been reported to play key roles during flower development in plants (Wu et al., 2006; Lee et al., 2010; Nag and Jack, 2010; Kim et al., 2011; Xia et al., 2012; Zhang et al., 2013; Spanudakis and Jackson, 2014). The suggested target gene of miR3980 is *pectinesterase*, which may be a key regulator in pollen fertility and grain filling. In the present study, we have confirmed the negative correlation between miR3980 and *pectinesterase* in expression (Figure 6A). These results suggest that heat stress can affect the expression of many miRNAs which are involved in flower development or fertility in rice. Based on these results, the module of the putative network of the seven heat-responsive miRNAs and their respective target genes involved in heat tolerance and flower development or fertility is presented in Figure 8. As flowering stage is a critical stage for plant fitness and crop yield (Huijser and Schmid, 2011), a detailed understanding of the involvement of miRNAs in heat tolerance at the flowering stage is essential for yield improvement in agricultural practice.

Like miRNAs, small interfering RNAs (siRNAs) have also been demonstrated to play important roles in stress tolerance (Khraiwesh et al., 2012). In our sequencing data, the group with 24 nt was the largest size group for the small RNAs. Since canonical heterochromatic siRNAs are 24 nt, this result implies that siRNAs may be also involved in rice heat tolerance. However, further study is needed to see if siRNAs participate in regulation of rice in response to heat stress.

### There are convergent and divergent mechanisms of miRNA in regulation of heat stress response in plants

In this study, we identified that 16 miRNAs of the 102 DE miRNAs were up-regulated whereas the others were all down-regulated by heat stress. This result is consistent with the previous analysis in *Brassica rapa* and *Populus tomentosa* that most identified miRNAs were down-regulated under heat stress (Yu et al., 2011; Chen et al., 2012). Interestingly, among the 102 DE miRNAs, 17 miRNA families including miR156, miR397, and miR398 have been demonstrated to be involved in heat stress response at the seedling stage in rice and other plant species (Xin et al., 2010; Jeong et al., 2011; Yu et al., 2011; Chen et al., 2012; Guan et al., 2013), further implying their important roles in heat stress response. The GO analysis of the target genes in the present study also showed that the most abundant GO items were metabolism, biological regulation, and biosynthesis, in keeping with the previous studies in wheat and Arabidopsis (Qin et al., 2008; Guan et al., 2013). These results indicated that there are common mechanisms of miRNAs in regulation of heat tolerance between different growth stages and among different plant species. However, compared with the previous study at the seedling stage (Xin et al., 2010; Jeong et al., 2011; Yu et al., 2011), we also identified different set of heat responsive miRNAs in the present study. For example, many miRNAs, such as miR156, miR159, miR160, and miR166, were up-regulated by heat stress in wheat (Xin et al., 2010), whereas these miRNAs were down-regulated in our study. MiR397b was induced by heat stress at the seedling stage in rice (Jeong et al., 2011) while it was down-regulated at the flowering stage. Moreover, 40 miRNAs were firstly reported to be involved in heat stress response in this study. These results indicated that there are different mechanisms of miRNAs in regulation of heat tolerance between different growth stages and among different plant species. Taken together, we can conclude that there are convergent and divergent mechanisms of miRNA in regulation of heat stress response in plants.

### The different expression patterns of miRNAs in GXN and HJX under heat stress may contribute to their contrasting heat tolerance

In previous studies, only one genotype is used to identify the heat stress responsive miRNAs in plants. To help better understand the mechanism of heat tolerance at flowering stage in rice, we used GXN and HJX, the two rice genotypes with contrasting heat tolerance for pairwise analysis of miRNAs. Our results revealed significant differences of miRNAs in expression between the two rice genotypes before and after heat stress. Firstly, a large number of conserved miRNAs (e.g., miR160, miR166, miR167, and miR168) exhibited higher expression level in the heat tolerant variety GXN over the heat sensitive variety HJX before heat stress. Secondly, more DE miRNAs were identified and the response of DE miRNAs to heat stress was much earlier in GXN than that in HJX. As shown in Table S7, 76 DE miRNAs were specifically expressed in GXN whereas only 17 DE miRNAs were specifically identified in HJX. As miRNAs exert their functions through targeting mRNA transcripts, more DE miRNAs in heat-tolerant variety GXN resulted in an increased transcriptome variation than that in the heat-sensitive variety HJX. Specifically, the number of target genes involved in biological process or tissue development was much larger in GXN over HJX. Thirdly, the co-regulated miRNAs under heat stress also exhibited differential expression patterns between GXN and HJX. Among the nine co-regulated miRNAs, we identified that miR396e-5p and miR396f-5p were significantly up-regulated in the tolerant variety under heat stress but were down-regulated in the sensitive variety, suggesting they may positively regulate heat tolerance. In contrast, the expression of miR159a.1, miR159b, and miR528-3p was repressed in the tolerant variety under heat stress but were induced in the sensitive variety, implying they may negatively regulate heat tolerance. Furthermore, the four miRNAs (miR164d, miR166i-3p, miR168a-3p, and miR397b) all exhibited the same suppressed expression pattern in both the tolerant and sensitive varieties after heat stress treatment, implying that they may act in fundamental responses to heat stress. Except for heat stress, previous reports have indicated that the four miRNAs were also involved in other abiotic stresses, such as drought and salinity (Kansal et al., 2015; Xie et al., 2015), More importantly, miR164 also showed an similar response to drought stress in both tolerant and sensitive varieties (Kansal et al., 2015), further supporting its persistent regulatory role in abiotic stresses. Based on these results, we deduced that these differences might partially account for their contrasting heat tolerance of the two rice varieties. Similarly, the earlier studies have demonstrated that the heat-resistant tomato (*Solanum lycopersicum L*.) cultivar (cv. 3042) exhibited higher basal expression of *HSP101* compared with the heat-susceptible cultivar (cv. 3017) and constitutive expression of *HSP101* could elevate the thermo-tolerance successfully (Katiyar-Agrawal et al., 2003; Pressman et al., 2007; Frank et al., 2009). Additionally, the previous study has indicated that the number of differentially expressed genes (DEGs) was higher in drought-tolerant rice than in drought-susceptible rice (Moumeni et al., 2011). Quicker and more efficient reversion of gene expression was also observed in chilling-tolerant genotype than in chilling-sensitive genotype (Zhang et al., 2012). These results together with our results suggest that higher basal expression of some important genes, more differentially expressed genes and quick response of the related genes may be the common features of tolerant genotypes over sensitive genotypes in response to abiotic stresses in rice.

### High-throughput sequencing combined with QTL mapping provides an efficient way to excavating candidate miRNAs

Though, RNA sequencing has been proved to be a powerful method in identifying miRNAs and their target genes associated with stress response (Xin et al., 2010; Jeong et al., 2011; Yu et al., 2011), it results in a large number of candidates. It is very difficult to pinpoint the miRNAs and genes that are important for the traits of interest. In the present study, we have identified 102 miRNAs that are significantly regulated by heat stress at the flowering stage. To screening for the important candidate miRNAs and genes that are involved in heat tolerance in rice, we employed RNA sequencing and QTL mapping. Two rice genotypes with contrasting heat tolerance were also used to help identify the candidate miRNAs and their target genes. By analyzing the significantly differentially expressed miRNAs between GXN and HJX under heat stress, together with analyzing the DE miRNAs whose targets are located in the QTL regions, we were able to successfully reduce the number of candidate miRNAs to 26 that belong to 6 miRNA families and identify 8 target genes within the QTL regions. These miRNA families include the three conserved miRNA families, miR166, miR169, and miR319, which have been confirmed to play pivotal roles in other stresses (Zhou et al., 2010; Ni et al., 2013; Li Y. et al., 2014). After differential expression analysis of the 8 target genes by RT-PCR, the number of candidate genes was further confined to four which showed differential expression between GXN and HJX under heat stress. Based on literatures and bioinformatics analysis, *LOC_Os12g42400, SGT1* and *pectinesterase* are the most possible targets associated with heat tolerance at the flowering stage in rice. We identified that the transcription of miR169r-5p was significantly up-regulated in both heat-tolerant variety GXN and heat-sensitive variety HJX at 1 and 6 h after heat stress treatment, but the expression levels in GXN were higher than that in HJX (Figure 6B). Accordingly, the transcription of its target gene *LOC_Os12g42400* was significantly down-regulated by heat stress treatment in heat-tolerant variety GXN, but its expression is almost not changed when subjected to heat stress in heat-sensitive variety HJX (Figure 6A). These results indicated the important roles of miR169r-5p and its target *LOC_Os12g42400* in heat tolerance. Further, the miR169r-5p overexpressing plants, in which the transcription of *LOC_Os12g42400* was significantly down-regulated, showed significantly higher spikelet fertility compared to wild type plants. Thus, the function of miR169r-5p on heat tolerance can be confirmed. Taken together, our results suggest that integrating RNA sequencing with QTL mapping is an efficient and cost-effective way to narrow down candidate miRNAs and target genes.

## Author contributions

QL conducted the quantitative qRT-PCR assay, transgenic functional confirmation, drafting the manuscript and proposal writing. TFY conducted the QTL analysis for heat tolerance and TY conducted the bioinformatics analysis. SZ, XW, JZ, XM, and JD participated in RNA extraction and quantitative qRT-PCR assays. BL conceived of the study, drafted proposal, and corrected manuscript.

## Funding

This research was supported by the National Youth Science Foundation of China (31201198), the Key Project of Guangdong Scientific and Technological Plan (2015B020231002), and the Guangdong Modern Agricultural Creation Team Project (2016LM2148).

### Conflict of interest statement

The authors declare that the research was conducted in the absence of any commercial or financial relationships that could be construed as a potential conflict of interest.
