# Supplementary material for: Integrating Small RNA Sequencing with QTL Mapping for Identification of miRNAs and Their Target Genes Associated with Heat Tolerance at the Flowering Stage in Rice
Source: Front Plant Sci. 2017 Jan 24;8:43. doi: 10.3389/fpls.2017.00043 (PMC5258760; doi:10.3389/fpls.2017.00043)
Supplement: Supplementary file 11 [file Image1.PDF]

Integrating RNA sequencing with QTL mapping for identification of miRNAs and their target genes associated with heat tolerance at the flowering stage in rice

Qing Liu[1,2 §], Tifeng Yang[1,2 §],Ting Yu[3 §], Shaohong Zhang[1,2], Xingxue Mao[1,2], Junliang Zhao[1,2], Xiaofei Wang[1,2], Jingfang Dong[1,2], Bin Liu[1,2*]

[1] Guangdong Key Laboratory of New Technology in Rice Breeding, Guangzhou 510640, China

[2] Rice Research Institute, Guangdong Academy of Agricultural Sciences, Guangzhou 510640, China

[3]Agro-biological Gene Research Center, Guangdong Academy of Agricultural Sciences, Guangzhou 510640, China

[§]Equally contributed to this work

Supplementary Figure. S1

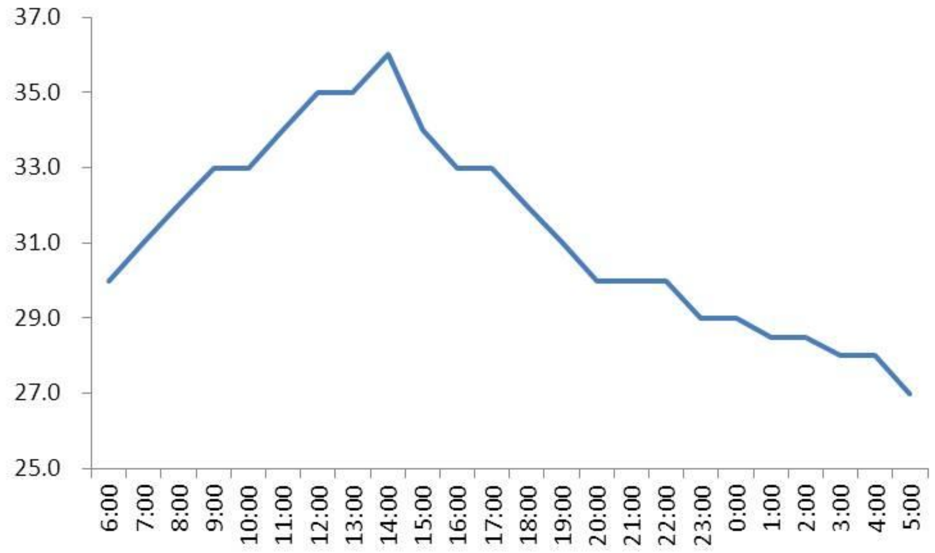

Figure S1. The concrete day and night temperature parameters used for heat tolerance evaluation.

Supplementary Figure. S2

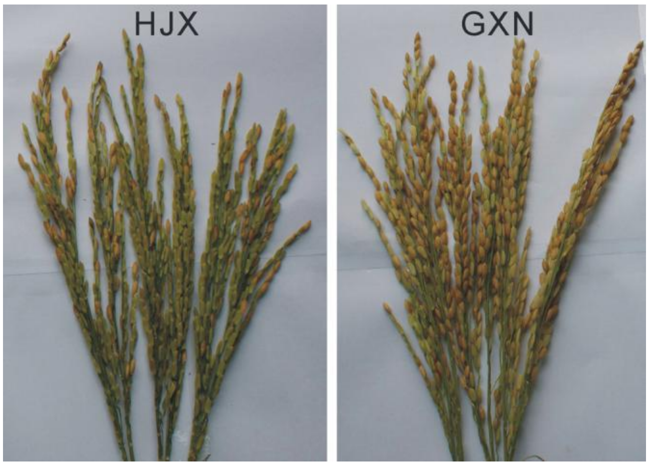

Figure S2. Phenotypic performance of GXN and HJX under heat stress treatment at the flowering stage.

Supplementary Figure. S3

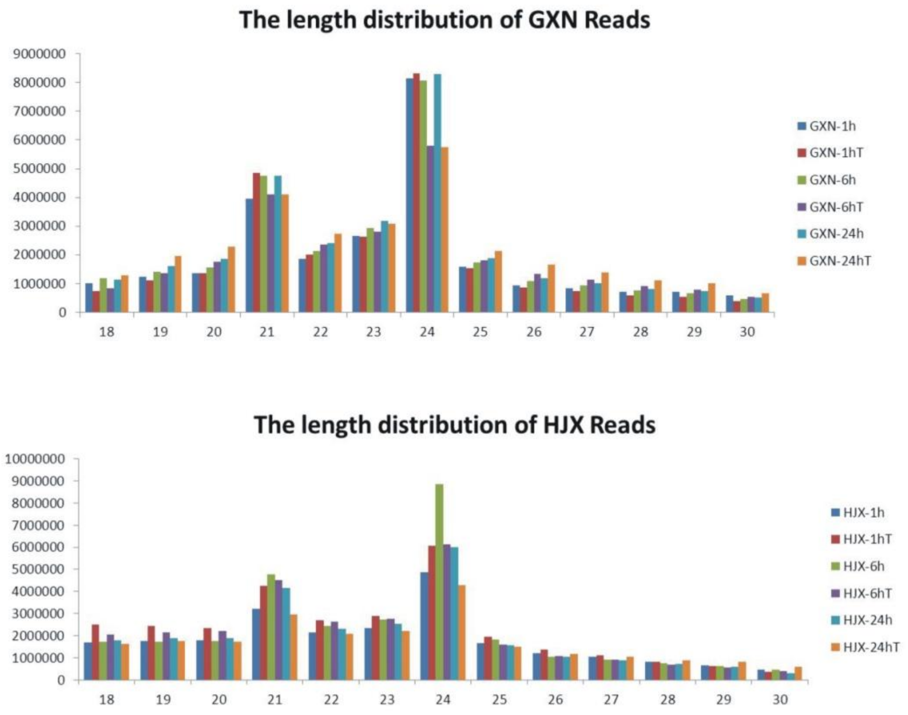

Figure S3. The length distribution of GXN and HJX reads.

Supplementary Figure. S4

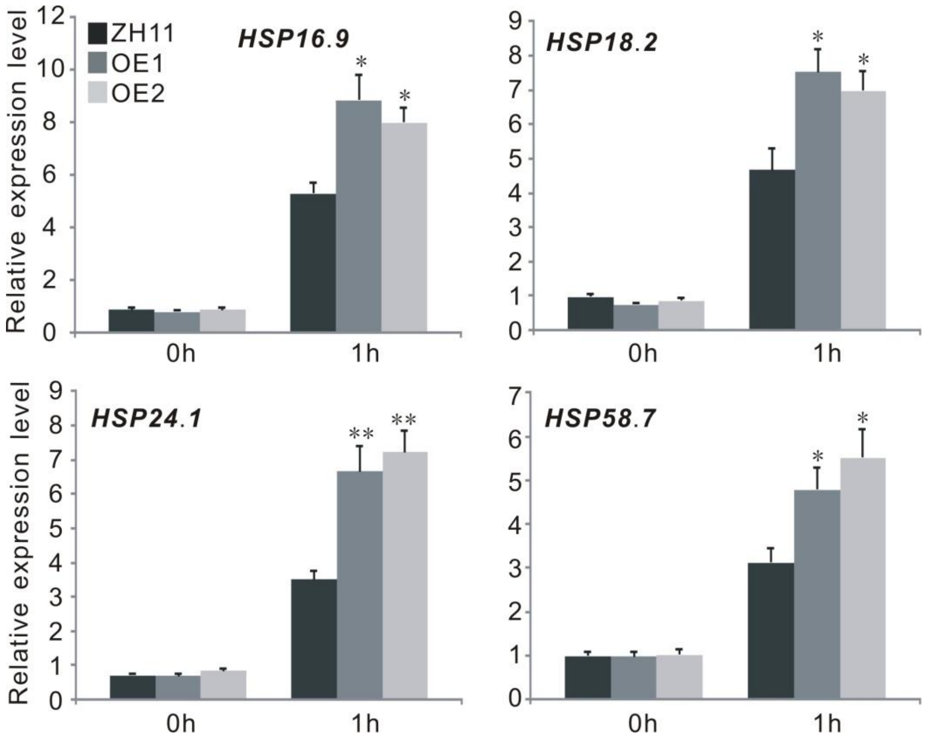

Figure S4. Transcription analysis of four heat shock proteins in ZH11 and miR169r-5p overexpressing plants under control and heat stress conditions at the flowering stage. Bars represent means (three replicates) ±SD, and asterisks indicate statistically significant differences compared with control (ZH11) (t test, $**P < 0.01$).
